# Disruption of *DELLA* or *ERECTA* suppresses the enlarged phloem phenotype caused by *hawaiian skirt* in the tomato cv. Micro-Tom

**DOI:** 10.5511/plantbiotechnology.26.0330a

**Published:** 2026-06-25

**Authors:** Michael D. Thomas, Ping-Wei Chen, Tarek El mestari, Fabien Lombardo

**Affiliations:** 1 Graduate School of Life and Environmental Sciences, University of Tsukuba, Tsukuba, Ibaraki 305-8572, Japan; 2 Global Innovation Joint-Degree Program, College of Medicine, National Taiwan University, Taipei 106319, Taiwan; 3 Université de Bordeaux, Collège Sciences et Technologies, Talence CEDEX 33405, France; 4 Faculty of Life and Environmental Sciences, University of Tsukuba, Tsukuba, Ibaraki 305-8572, Japan

**Keywords:** pedicel, phloem, tomato, vascular tissue, xylem

## Abstract

Modulation of plant vascular tissues is of interest for crop improvement because xylem and phloem are responsible for wood production and sugar transport across the plant, respectively. Previous studies have shown that *DELLA* and *ERECTA* (*ER*) regulate vascular tissue development, as their respective mutants exhibit enlarged xylem and disorganized vascular tissue. The tomato *hawaiian skirt-1* (*hws-1*) mutant accumulates microRNAs and displays a strongly enlarged phloem together with slightly delayed xylem development in pedicels. In this study, *della* and *er* mutant lines were crossed with *hws-1* to evaluate the effect of disrupted *DELLA* and *ER* function on the large-phloem phenotype of *hws* in the resulting double mutants. Measurement of miRNA164 levels, a microRNA that accumulates in *hws*, revealed that its abundance returned to wild-type levels in the double mutants, indicating that the effects of *della* and *er* mutations dominate over *hws*. Consistently, plant architecture and vascular phenotypes in the double mutants closely resembled those of the *della* and *er* single mutants, and the characteristic phloem enlargement of *hws* was absent. These results indicate that *DELLA* and *ER* functions are required for the manifestation of the *hws* large-phloem phenotype in tomato cv. Micro-Tom and that these genes are largely epistatic to *hws* for the majority of the morphological traits examined.

## Introduction

Plant radial growth increases the cross-sectional area of vascular tissues and represents a promising target for improving plant productivity. Radial expansion of xylem produces lignified or woody tissues essential for timber production, while radial expansion of phloem can enhance the transport capacity for carbohydrates and other metabolites directed toward developing fruits (Beuchat et al. 2020; Cornelis and Hazak 2022). Radial growth is regulated by plant hormones such as gibberellins (GA), which promote xylem expansion at the expense of the phloem during the second phase of this developmental process (Ben-Targem et al. 2021; Ragni et al. 2011; Smet and De Rybel 2016). The GA response is mediated by DELLA proteins, which act as integrative hubs that mediate crosstalk with multiple hormones, such as auxin, brassinosteroids, ethylene, jasmonate, and abscisic acid, thereby coordinating plant development and stress responses. DELLA proteins repress growth-related genes, and GA binding to the GID1 receptor relieves this repression by promoting DELLA degradation through the ubiquitin-26S proteasome pathway (Hedden and Sponsel 2015). In vascular tissues, DELLAs physically interact with the transcription factor KNAT1/BREVIPEDICELLUS and inhibit its binding to target promoters, thereby repressing transcriptional programs required for xylem fiber differentiation and secondary cell wall formation (Felipo-Benavent et al. 2018). Consequently, DELLA accumulation suppresses lignification, phenylpropanoid metabolism, and the expression of master regulators of fiber differentiation. DELLAs also regulate dynamics of the cambium, the meristem driving vascular radial growth, by interacting with AUXIN RESPONSE FACTORS ARF6 and ARF8, which are re-quired for GA-dependent xylem expansion and repression of phloem proliferation (Ben-Targem et al. 2021). Through DELLA-ARF interactions, high DELLA levels maintain cambial activity, delay cambium senescence, and bias vascular output toward continued phloem production rather than xylem expansion. Upon GA-induced DELLA degradation, KNAT1, ARF6, and ARF8 are released, promoting xylem production, differentiation of xylary fibers, repression of phloem proliferation, and progression toward cambium senescence.

Functionally related to *DELLA*, the *ERECTA* (*ER*) gene encodes a receptor-like kinase that regulates meristematic activity, vascular tissue development and gibberellin biosynthesis (Sarnowska et al. 2023; Shpak et al. 2004). In tomato, *er* mutants display a plethora of phenotypic alterations that collectively result in a compact and dwarf architecture, characterized by markedly reduced plant height due to shortened internodes and tightly clustered inflorescences (Chen et al. 2024; Kwon et al. 2020). These phenotypes contrast sharply with those observed in *della* mutants with impaired repression function, which exhibit increased internode length, thinner stems, and fewer leaflets with less serration (Jasinski et al. 2008). Alterations in vascular organization have also been reported in *er* lines of *Brachypodium distachyon* (Sakai et al. 2021). These vascular phenotypes include disrupted vascular bundles, amphivasal configurations in which phloem is surrounded by xylem, misshapen sieve elements, altered phloem area, disorganized xylem bundles, and an increased number of vascular bundles. Loss of *ER* function additionally leads to reduced lignin and polysaccharide deposition in stem cell walls. Together, these observations indicate that *ER* plays an important role in vascular patterning and proliferation, as well as secondary cell wall deposition.

A few genes other than *ER* and *DELLA* have been directly linked to such pronounced phenotypic changes in vascular tissue. Notably, the *hawaiian skirt* (*hws*) mutant in tomato cv. Micro-Tom exhibits a strong increase in phloem area and a slightly delayed expansion of xylem (Lombardo et al. 2021, 2025). Mutant lines of *hws* were first described in *Arabidopsis thaliana*, revealing a role for the gene in microRNA (miRNA) regulation (Lang et al. 2018; Mei et al. 2019). In tomato, the weak *hws-1* allele shows several phenotypic alterations, including reduced leaf serration, partial fusion of leaflets, reduced pollen viability, and taller plants with thicker stems and pedicels (Damayanti et al. 2019). These traits are associated with increased levels of several miRNAs, although it remains unclear which of the miRNA species accumulating in *hws* are responsible for its pronounced vascular phenotype (Lombardo et al. 2025). The increased sugar content measured in *hws-1* fruits correlates with the enlarged phloem area, highlighting the mutant’s potential for crop improvement (Lombardo et al. 2021). Given the potential of *della*, *er*, and *hws* as targets for crop improvement, clarifying the respective influences of *della* and *er* on *hws* in the regulation of vascular tissue development is therefore of particular interest. To our knowledge, the vascular tissue phenotype of *della* and *er* loss-of-function mutants has not been described in tomato. The present study reports the whole-plant and pedicel vascular phenotypes of single and double mutant lines, providing evidence for epistasis of *della* and *er* over *hws*. Epistasis is defined here phenotypically, in that the vascular and architectural traits of the double mutants more closely resemble those of the *della* or *er* single mutants than those of *hws-1*.

## Materials and methods

### Plant material and cultivation

Tomato (*Solanum lycopersicum*) *hws-1* (TOMJPE8986), *er* (TOMJPE5066-1), and *dellaS485L* (TOMJPE7633-1) mutant lines were isolated from an ethyl methanesulfonate (EMS) mutagenized population of the cultivar “Micro-Tom” generated by the National BioResource Project (Saito et al. 2011). The *hws-1* and *er* mutants were previously described by Chen et al. 2024 and Damayanti et al. 2019, respectively, whereas dellaS485L is characterized in the present study. For simplicity, *dellaS485L* is hereafter referred to as *della*. For the single mutants, a BC_3_ line was used for hws-1, whereas BC_1_ lines were used for both *er* and *della*. Each mutation was confirmed by Sanger sequencing in homozygous F_3_ or later single-mutant populations and in F_2_ double-mutant populations. Plants were grown in rockwool and irrigated with Ōtsuka House solutions No. 1 and No. 2 (OAT Agrio Co., Ltd., Tokyo, Japan). Growth conditions consisted of a 16 h light/8 h dark photoperiod provided by LED lighting with a photosynthetically active radiation (PAR) intensity of 230 µmol m^−2^ s^−1^. All six lines, namely WT, *hws-1*, *dellaS485L* (hereafter referenced as *della* for simplicity), *er*, and the two double mutants *hws-1/della* and *hws-1/er*, were grown in parallel.

### RT-qPCR

Pedicel tissue at nine days after anthesis was collected by excising pedicel segments between the abscission zone and the calyx and immediately snap-freezing them in liquid nitrogen. When the abscission zone was not clearly identifiable, segments were excised approximately 1 mm above the calyx.

Individual pedicel segments were ground using a mortar and pestle, and total RNA was extracted with a High Pure miRNA Isolation Kit (Roche, Basel, Switzerland) following the manufacturer’s instructions (single-column protocol). Measurements were performed using five biological replicates.

cDNA was synthesized from 30 µg of total RNA using SuperScript IV VILO Master Mix with ezDNase enzyme (Life Technologies, California, USA). The resulting cDNA was diluted twofold prior to RT-qPCR analysis.

RT-qPCR reactions were performed using a CFX96 Real-Time System (Bio-Rad, California, USA) with TB Green Premix Ex Taq II (Takara Bio Inc., Shiga, Japan) in a two-step amplification protocol according to the manufacturer’s instructions. miRNA levels were quantified using the two-tailed RT-qPCR method described by Androvic et al. 2017. Gene expression levels were normalized to the *SAND* reference gene (*Solyc03g115810*) as described by Expósito-Rodríguez et al. 2008. Primer sequences are listed in Supplementary Table S1.

### Microscopy

Pedicels of 20 days after anthesis or older, after rapid vascular tissue development stage (Lombardo et al. 2025), were excised from plants and immediately sectioned using a VT1200S vibratome (Leica Microsystems, Wetzlar, Germany) at a thickness of 150 µm. Sections were obtained approximately halfway between the abscission zone and the fruit, corresponding to the region of smallest pedicel diameter. For light microscopy, sections were stained with 0.05% toluidine blue for approximately 1.5 min, briefly rinsed with distilled water, and observed using an Olympus BX53 light microscope (Olympus Corporation, Tokyo, Japan). Images were captured using cellSens Standard 1.6 imaging software [Olympus; http://www.olympus-global.com (Accessed Dec 27, 2025)].

The vascular tissue area was measured manually in QuPath (Bankhead et al. 2017) using the wand tool. Automatic segmentation was not used because it did not reliably delineate tissue boundaries in these samples. Tissues were identified based on toluidine blue staining intensity and hue, anatomical position, and general cellular morphology. Images were adjusted for brightness, sharpness, and color balance using Adobe Photoshop (Adobe Inc., San Jose, CA, USA).

### Statistical analysis

Statistical analysis was conducted using R v4.5.1 (R Core Team 2025), in RStudio (Posit Team 2024). Vascular tissue ratios were analyzed using the non-parametric Kruskal–Wallis test due to differences in sample size and heteroscedasticity (Bartlett’s test, *p*<0.05). A Dunn’s test was used for pairwise comparisons. Letters denote statistically significant differences between groups at *p*=0.05. RT-qPCR expression data was normalized to WT *SAND* expression. RT-qPCR data were evaluated for normality and equal variance using a Q-Q plot and Bartlett’s test and did not violate the assumptions of normality and equal variance. A one-way ANOVA was used for statistical analysis, and Tukey’s post-hoc comparisons were conducted when the ANOVA was significant. Letters denote statistically significant differences between groups at *p*=0.05.

## Results

### Plant architecture and leaf phenotype

**Figure 1:**
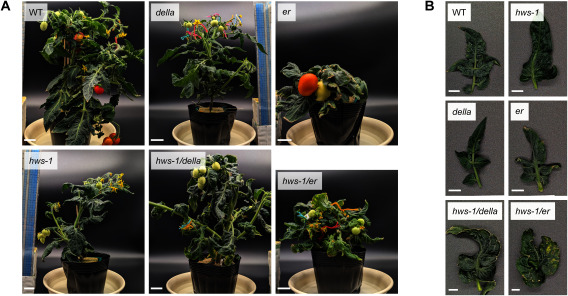
Figure 1. Mutant lines display altered plant architecture and leaf morphology. (A) Mutant lines exhibit either open or compact architecture compared with WT. (B) Mutant leaves show varying degrees of curling, elongation, leaflet fusion, and serration. Bars indicate 2 cm.

The *hws-1* mutant exhibits the characteristic increase in plant height, visually thicker stems and pedicels, and a strong reduction in leaf serration as previously reported in Damayanti et al. 2019 and Nagata et al. 2021 (Figure 1A, B).

**Figure 2:**
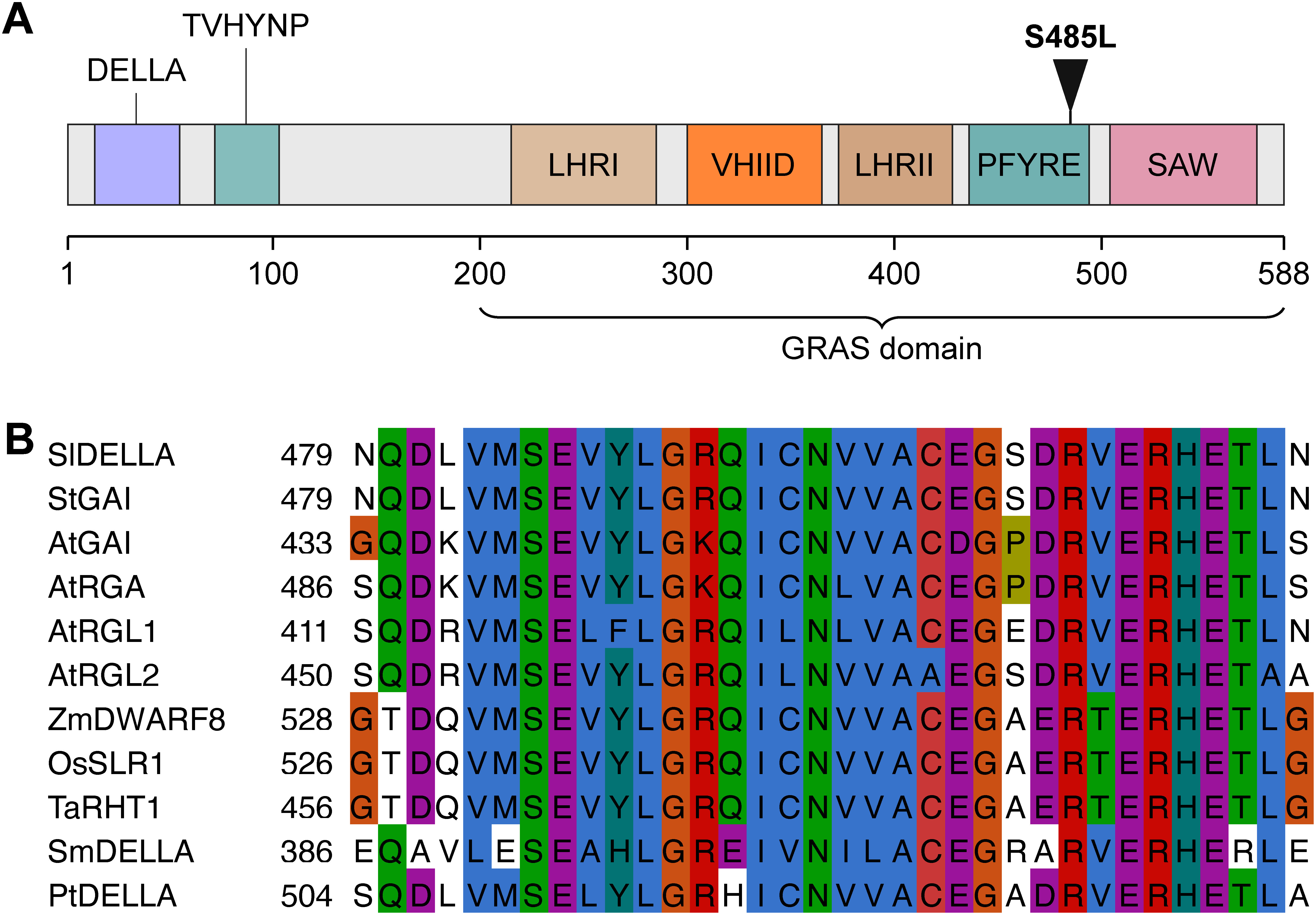
Figure 2. Position of the mutation in the *della* line used in this study. (A) Schematic representation of the DELLA protein. (B) Partial representation of the PFYRE motif. Black triangles indicate the conserved Ser485 residue, which is substituted by Leu in the *della* mutant line used in this study.

The *della* line used in this study was isolated based on its increased height by the late Professor Ariizumi Tohru, who sequenced it and generated BC_1_ populations (personal communication). We confirmed that the line carries a mutation in the PFYRE motif, which is part of the GRAS domain of the protein (Figure 2A, B). The mutation replaces the conserved serine at position 485 with a leucine (Figure 2B). Mutation affecting conserved residues in the PFYRE motif have been shown to disrupt the interaction with histone H2A, which is required for the repression of GA-responsive genes (Huang et al. 2023, 2024), suggesting that S485L has a similar effect. The mutant exhibits increased internode length (18.7 mm compared to 16 mm in WT) and simplified, less serrated, elongated leaflets compared to WT (Figure 1A, B), consistent with previous descriptions of *della* mutants showing enhanced gibberellin responses (Carrera et al. 2012; Jasinski et al. 2008). Both the *er* and *hws-1/er* lines produce very short plants with compact inflorescences. All morphological traits previously described for the *er* mutant in Chen et al. 2024, namely reduced plant height, shorter internode and pedicel length, increased stem diameter, and lower seed number, were also observed in the double mutant, which differs only in the presence of the leaflet fusion characteristic of *hws-1* (Figure 1B). A comparable pattern is observed in the *hws-1/della* mutant, which is indistinguishable from the *della* line except for the *hws-1*-associated leaf defects. Together, these observations indicate that the effects of the *hws* mutation are minor compared with the stronger developmental alterations caused by either *er* or *della*. An exception to this pattern was observed during reproduction, where the *hws-1/della* double mutant exhibited a markedly increased rate of flower abortion compared with the other lines (Supplementary Table S2).

### miRNA164 abundance

**Figure 3:**
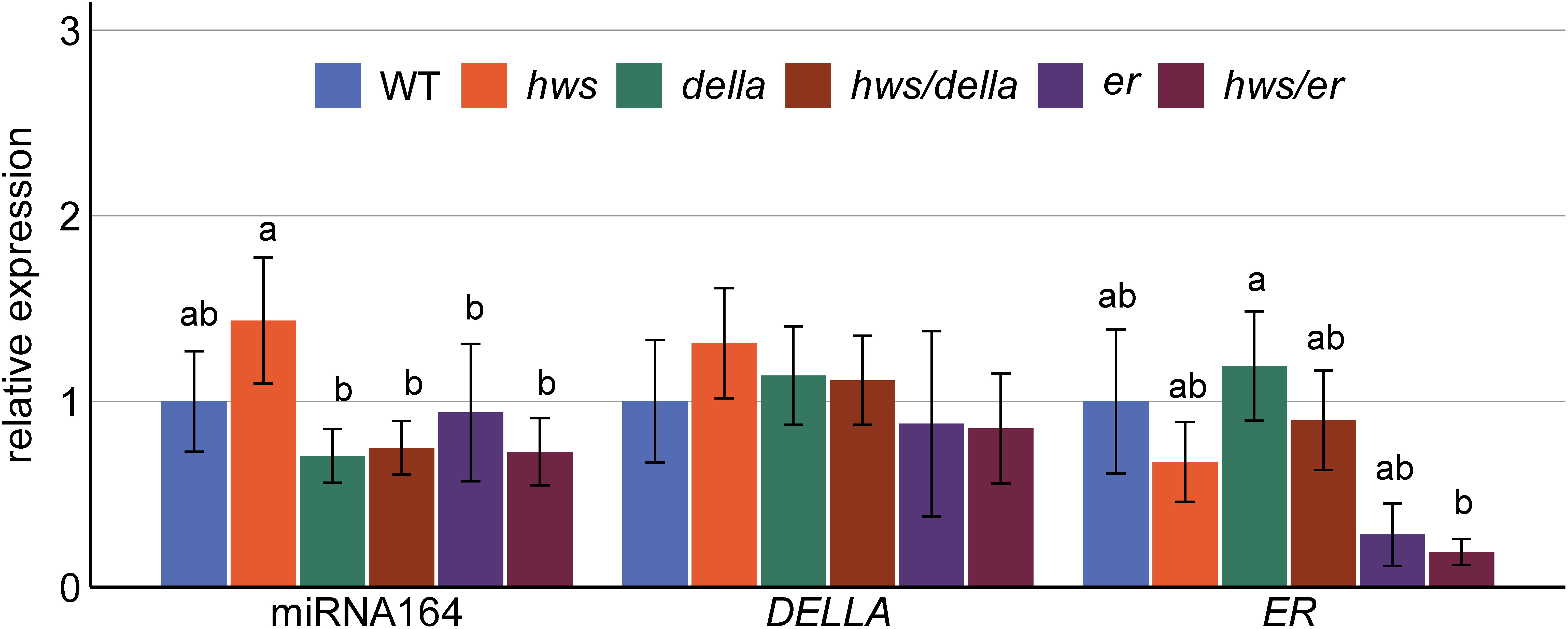
Figure 3. Relative expression of miRNA164, *DELLA* and *ER* across lines. RT-qPCR expression data were normalized to WT SAND expression. Statistical analysis was performed using one-way ANOVA with Tukey’s post-hoc test. Different letters indicate significant differences at (*p*=0.05).

As noted in the introduction, several miRNAs have been reported to accumulate in *hws* mutants. Among these, miRNA164 accumulation has been confirmed in pedicels and flowers of *hws-1* tomato, supporting its use as a reliable indicator of *hws* molecular activity (Damayanti et al. 2019; Lombardo et al. 2025). Accordingly, miRNA164 abundance was quantified by RT-qPCR in double mutants carrying *hws* to assess whether miRNA accumulation is maintained (Figure 3). Expression of *DELLA* and *ER* was also investigated in all lines. Consistent with Lombardo et al. 2025, miRNA164 shows a trend toward increased abundance in *hws-1* compared to WT (*p*=0.13). Because RNA was extracted from whole pedicel tissue, differences in miRNA164 accumulation specific to vascular tissues may be partially diluted, which could account for the lack of statistical significance. All other lines display significantly lower levels of miRNA164. Expression of *ER* is only significantly different between *della* and *hws-1/er* lines, with all other lines showing non-significant variation. The *er* single mutant exhibits reduced *ER* expression similar to that of *hws-1/er*; however, this reduction is not statistically significant compared with the other genotypes. *DELLA* expression is not significantly different among genotypes.

### Vascular phenotype

Cross-sections of pedicels of 20 days after anthesis or older were examined under the microscope after toluidine blue staining. This allowed visual identification of phloem and xylem tissues. Cross-sectional area was measured after delineation with the QuPath segmentation software (Bankhead et al. 2017, Figure 4).

**Figure 4:**
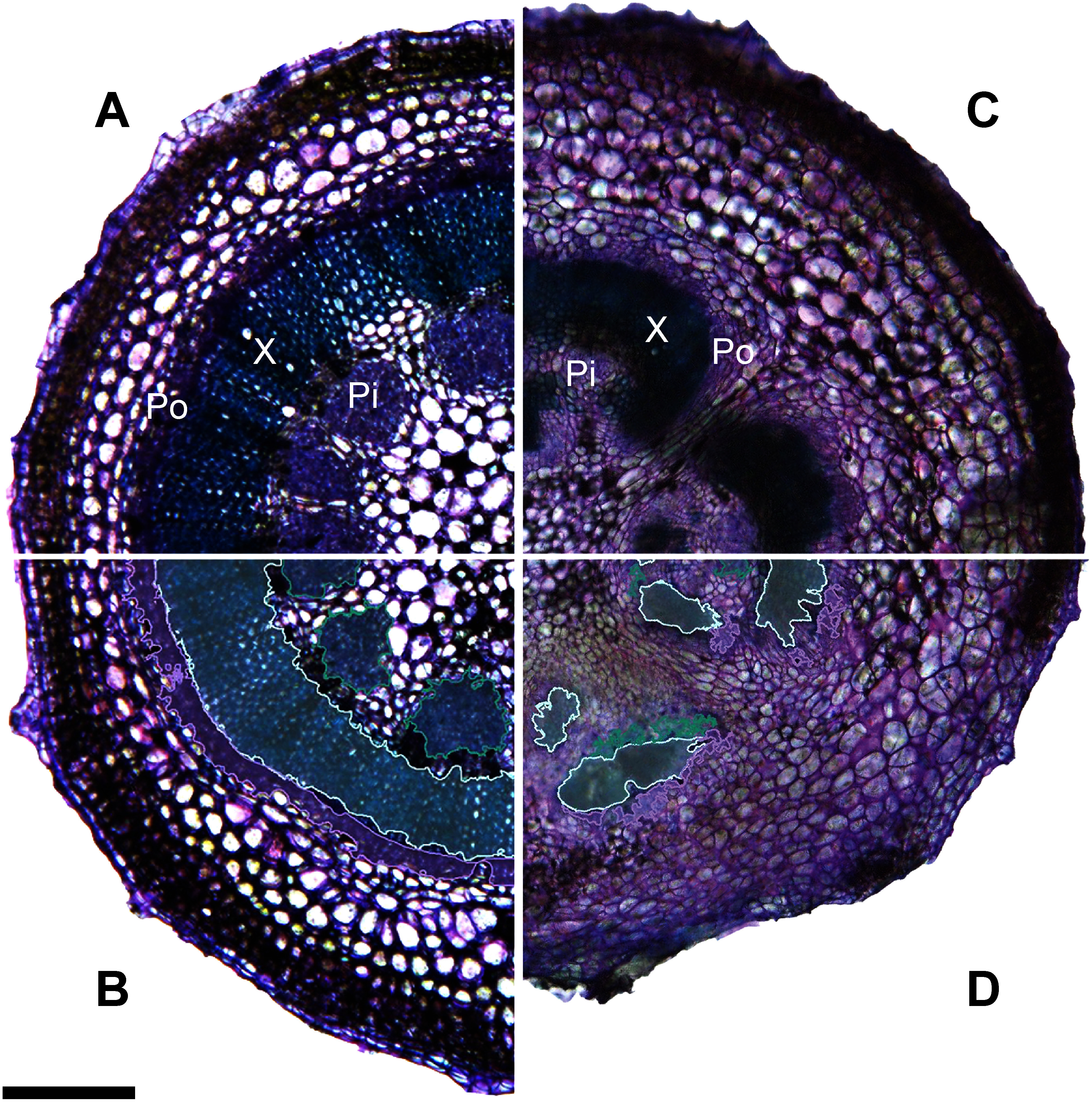
Figure 4. Vascular disorganization in pedicels of *hws-1/er* compared to WT. WT pedicel cross-section (A, B) is shown on the left and compared with a cross-section of a *hws-1/er* pedicel (C, D) on the right. Delineated tissues are highlighted in the lower panels (B, D). Outer and inner phloem tissues are indicated as Po and Pi, respectively, and xylem is indicated as X. The scale bar represents 200 µm.

**Figure 5:**
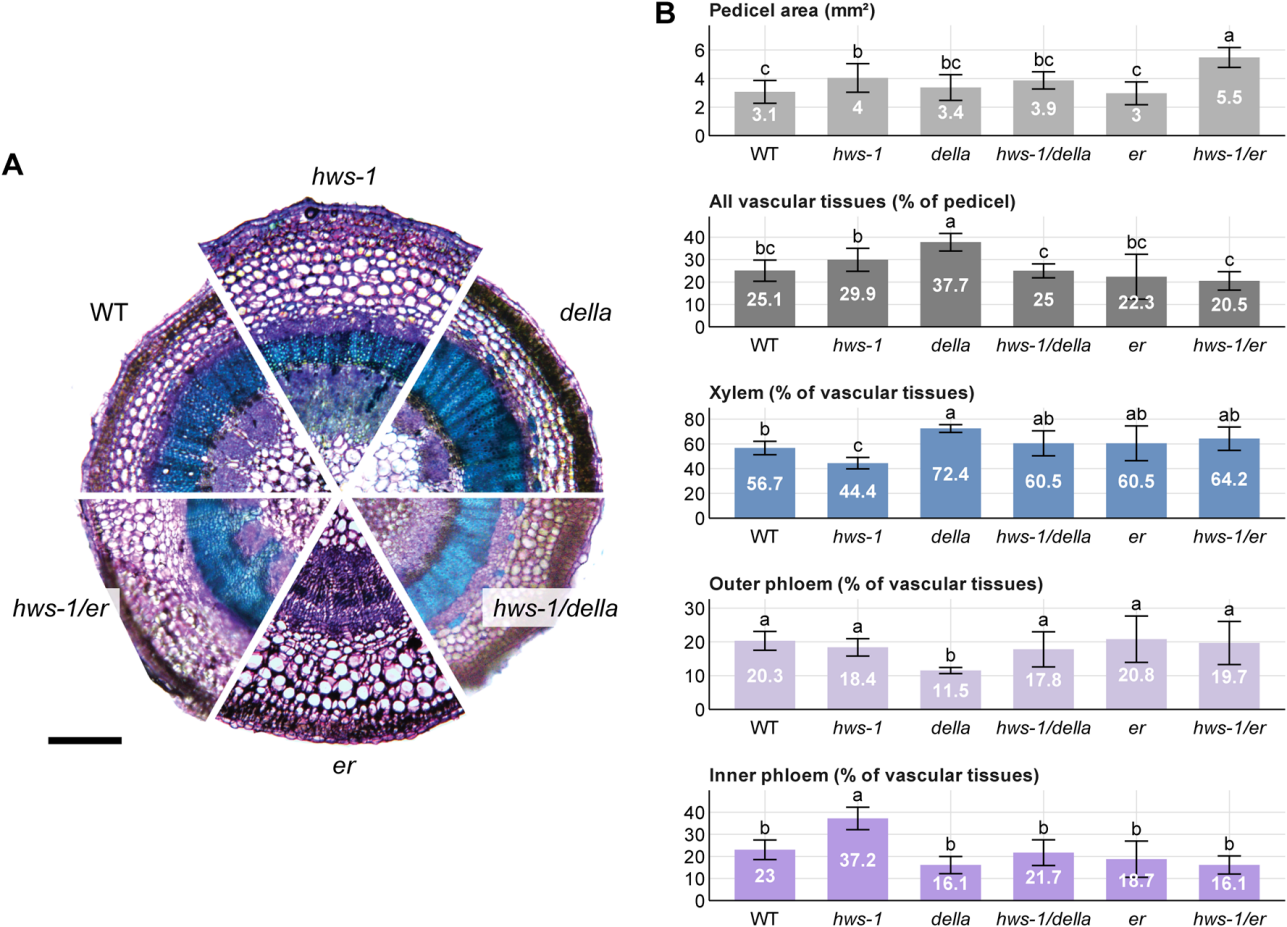
Figure 5. Vascular tissues of mutant and WT pedicels. (A) Representative pedicel cross-sections showing differences in vascular tissue organization. Bar indicates 200 µm. (B) Proportions of inner phloem, outer phloem, and xylem relative to total vascular tissue area (%), and mean pedicel area (mm^2^) across genotypes. Data analyzed using Kruskal–Wallis test and Dunn’s post-hoc comparison. Letters represent significant groups (*p*<0.05). Bars represent the standard deviation.

As reported previously, *hws-1* pedicels display a larger cross-sectional pedicel area (PA) than WT pedicels (Damayanti et al. 2019; Lombardo et al. 2025, Figure 5A). This increase in PA corresponds to an increased vascular tissue area, which constitutes 29.9±5.13% of total PA in *hws-1*, representing the second highest vascular tissue area (%PA) among the genotypes described (Figure 5B). Xylem comprises 44.4±4.56% of the vascular tissue (%VT), representing the lowest relative xylem area among the genotypes examined. Instead, the majority of the vascular tissue area in *hws-1* is composed of phloem, making it unique among the genotypes described here. Inner phloem contributes more than outer phloem to the vascular tissue in *hws-1*, with 37.2±5.10%VT consisting of inner phloem and 18.4±2.60%VT consisting of outer phloem. Outer phloem area is not significantly different from that of the other genotypes.

*della* pedicels show the highest vascular tissue area at 37.7±3.94%PA, with a PA not signifi-cantly different from that of WT or *hws-1*. Xylem represents 72.4±3.12%VT, making the relative xylem area significantly larger in *della* pedicels compared to the other genotypes, consistent with the role of *DELLA* in repressing xylem differentiation (Ben-Targem et al. 2021; Felipo-Benavent et al. 2018). Conversely, *della* plants show the lowest relative phloem area among the geno-types surveyed. Both the lowest inner and outer phloem areas occur in *della* pedicels, at 16.1±3.87%VT and 11.5±0.89%VT, respectively. Outer phloem area is significantly lower than in the other genotypes.

Vascular tissue area (%VT) in *er* pedicels is not significantly different from that of WT pedicels, but is significantly lower than that of both *hws-1* and *della*. *er* cross-sections often display abnormal vascular tissues, such as the absence of lignified cells typically indicative of xylem tissue. When vascular tissue development appears normal in *er* pedicels, xylem area tends to be relatively high. This dimorphism results in the highest variation in relative xylem and phloem areas among the genotypes examined. On average, 60.5±10.11%VT corresponds to xylem, 18.7±8.18%VT to inner phloem, and 20.8±6.86%VT to outer phloem. Compared with WT, *er* pedicels show a higher average xylem area, although this difference is not significant, likely due to the high variability observed in *er* pedicels.

Double mutant *hws-1/della* pedicels display a vascular tissue area similar to WT and signifi-cantly lower than that of *della* and *hws-1*, at 25.0±3.07%PA. *hws-1/della* pedicels also show a non-significant but higher average inner phloem area (21.7±5.77%VT) compared with *della* single mutants, but significantly lower inner phloem area than *hws-1*. Outer phloem area is significantly larger in *hws-1/della* double mutants relative to *della* single mutants, at 17.8±5.16%VT. These significant differences in vascular tissue phenotypes when compared with both *hws-1* and *della* suggest interactive effects that limit the phenotypic impact of both genes.

Similar to *er* single mutant pedicels, *hws-1/er* double mutant pedicels often display highly disorganized vascular tissues (see also Figure 4C, D), although this appears to occur more frequently and more severely (anecdotal observation, not quantified). Observed phenotypes include complete disruption of the xylem ring, allowing inner and outer phloem to connect, and the presence of inner phloem within xylem tissue. Despite having the largest PA, *hws-1/er* double mutants display the lowest average vascular tissue area among all genotypes, at 20.5±4.13%PA, although this value is not significantly different from the other lines except *hws-1* and *della*. Inner phloem tissue comprises 16.1±4.07%VT in *hws-1/er* double mutants, which is significantly smaller than in *hws-1* single mutant pedicels. Outer phloem tissue in *hws-1/er* is similar to that in WT, *hws-1*, and *er* pedicels, at 19.7±6.43%VT, suggesting no significant impact on outer phloem area in the double mutant line. The higher frequency of vascular tissue disorganization and the significant phenotypic differences relative to *hws-1* suggest interactive effects that also limit the single mutant phenotypes in the *hws-1/er* double mutant.

## Discussion

Vascular tissue size is a major determinant of sugar and water transport capacity (Konrad and Roth-Nebelsick 2023; Patrick 2013). Larger pedicels can accommodate larger vascular tissues, and pedicel cross-sectional area has been correlated with fruit yield under both normal and stress conditions (Savage et al. 2015; Simon et al. 2022). Genes regulating vascular tissue development are therefore of interest for modulating plant growth parameters. In the present study, we show that the ratio between vascular tissue area and total pedicel area (%PA) can vary substantially among genotypes within the Micro-Tom cultivar. For instance, *della* and *hws-1/er* exhibit similar vascular tissue area but markedly different pedicel sizes (Figure 5B).

The overall morphology of *hws-1/della* and *hws-1/er* plants differs from that of *della* and *er* single mutants primarily in leaf shape, with leaves exhibiting features characteristic of each additional mutation (Figure 1). This observation suggests that although the *hws-1* mutation affects plant phenotype, the effects of *della* or *er* mutations are dominant in determining morphology. A similar pattern occurs in pedicel cross-sections, where the characteristic large-phloem phenotype of *hws-1* was either completely or partially suppressed in the *hws-1/er* and *hws-1/della* lines, respectively (Figure 5B).

In *Brachypodium distachyon*, loss of *ER* function leads to vascular disorganization (Sakai et al. 2021), and several studies have identified *ER* function as necessary for normal organ development (Jiang et al. 2022; Shpak 2013; Tameshige et al. 2017). It should be noted that, as in the *Bder* mutant, lignin deposition in xylem cells is most likely reduced in the tomato counterpart. This reduction likely affects the staining intensity of toluidine blue in our sections, making precise measurement of xylem tissue in the *er* mutant challenging. Nevertheless, clear vascular disorganization occurs in our experiments, and this effect appears amplified in the *hws-1/er* double mutant (Figure 5A, B).

The influence of *hws-1* is also observed in the *hws-1/della* double mutant, with the inner phloem area being intermediate between that of the WT and the *della* single mutant. Additionally, high flower abortion rate observed in the *hws-1/della* double mutant may indicate a synthetic interaction between GA signaling and the HWS-associated pathway.

The enlarged phloem observed in *hws-1* has been associated with miRNA accumulation, although the specific miRNA species responsible for this phenotype remain unclear (Lombardo et al. 2025). miRNA164 was previously reported to accumulate in *hws-1* (Damayanti et al. 2019) but not in *della* or *er* mutants (Figure 3), and its accumulation in any of the double mutants would therefore suggest that the *hws* pathway remains active. In our results, however, miRNA164 expression was elevated only in the *hws* single mutant, indicating that this pathway is not strongly active in the double mutants. It is possible that miRNAs other than miRNA164 accumulate in these lines. Furthermore, miRNA accumulation in *hws* is likely restricted to vascular tissues (Lombardo et al. 2025), and differences in abundance may be attenuated in our measurements because RNA was extracted from whole pedicel tissue, including parenchyma and epidermal cells. Nevertheless, the absence of detectable miRNA164 accumulation in the double mutants is consistent with the relatively minor *hws*-associated phenotypic effects observed at both the morphological and vascular tissue levels in these lines.

The reduced *ER* expression observed in both the *er* single mutant and the *hws-1/er* double mutant suggests that the stop-inducing mutation destabilizes the truncated *ER* messenger RNA (Chen et al. 2024). In contrast, *DELLA* expression levels did not differ significantly among genotypes. The altered phenotype of the *della* mutant is therefore attributable to impaired protein function caused by disruption of the PFYRE domain (Figure 2A), which prevents *DELLA* from repressing GA-responsive genes even under low GA conditions.

In summary, this study indicates that the GA-related genes *DELLA* and *ER* act predominantly epistatically to the HWS-mediated miRNA pathway, highlighting their central role in vascular tissue development. This epistasis is defined at the phenotypic level and does not imply a strict upstream-downstream molecular relationship. Because *ER* also modulates the expression of several auxin biosynthesis genes (Chen et al. 2024), future studies aimed at disentangling the hormonal interactions among auxin, GA, and other phytohormones will be important for improving the understanding of vascular tissue development and its potential applications in enhancing plant productivity.

## Abbreviations

ER: ERECTA
HWS: HAWAIIAN SKIRT
miRNA: microRNA
PA: pedicel area
VT: vascular tissue
WT: wild-type

## Accession numbers

*DELLA: Solyc11g011260*; *ERECTA: Solyc08g061560*; *HAWAIIAN SKIRT: Solyc01g095370*.

## Description of supplementary files

Supplementary Table S1. Lists the primer sequences used in this study.

Supplementary Table S2. Reports the flower abortion rates observed in all studied lines.
