# Supplementary material for: Disruption of DELLA or ERECTA suppresses the enlarged phloem phenotype caused by hawaiian skirt in the tomato cv. Micro-Tom
Source: Plant Biotechnol (Tokyo). 2026 Jun 25;43(2):201–8. doi: 10.5511/plantbiotechnology.26.0330a (PMC13324320; doi:10.5511/plantbiotechnology.26.0330a)
Supplement: Supplementary Data [file plantbiotechnology-43-2-26.0330a-s001.pdf]

Supplementary files

Supplementary Table S1. Lists the primer sequences used in this study.

Name	Sequence (5′–3′)	Purpose	Reference
PR60	TGCTTCTCCACTATCCTCTCCAG-GTACAGTTGGTACCTGTCTCCAC-TTTGCAC	two-tailed primer for quantification of miRNA164	design method in Androvic et al. (2017)
PR61	TGCTTCTCCACTATCCTCTCC	RT-qPCR of miRNA164 (forward)	
PR62	TGGAGAAGCAGGGCACGT	RT-qPCR of miRNA164 (reverse)	
PR213	ACAACAATTTATCAGGCGATG	RT-qPCR of *ER* (forward)	Chen et al. 2024
PR214	CTAGAGAAATTATTGCCGGTT	RT-qPCR of *ER* (reverse)	
PR215	TGATGCGACTATACTTGATATAAG	RT-qPCR of *DELLA* (forward)	Carrera et al. 2012
PR216	GGGTTAATCTGTTTAATAGAGTTC	RT-qPCR of *DELLA* (reverse)	
PR217	TTGCTTGGAGGAACAGACG	RT-qPCR of *SAND* (forward)	Expósito-Rodríguez et al. 2008
PR218	GCAAACAGAACCCCTGAATC	RT-qPCR of *SAND* (reverse)	

Supplementary Table S2. Reports the flower abortion rates observed in all studied lines.

**Line**	**Flower abortion (n/total)**	**Flower abortion rate (%)**
WT	18/192	9.4
hws-1	7/82	8.5
er	2/19	10.5
hws-1/er	4/38	10.5
della	2/26	7.7
hws-1/della	36/44	81.8

References

Androvic P, Valihrach L, Elling J, Sjoback R, Kubista M (2017) Two-tailed RT-qPCR: A novel method for highly accurate miRNA quantification. *Nucleic Acids Res* 45: e144

Carrera E, Ruiz-Rivero O, Peres LEP, Atares A, Garcia-Martinez JL (2012) Characterization of the procera tomato mutant shows novel functions of the SlDELLA protein in the control of flower morphology, cell division and expansion, and the auxin-signaling pathway during fruit-set and development. *Plant Physiol* 160: 1581–1596

Chen D, Xu Y, Li J, Shiba H, Ezura H, Wang N (2024) ERECTA modulates seed germination and fruit development via auxin signaling in tomato. *Int J Mol Sci* 25: 4754

Expósito-Rodríguez M, Borges AA, Borges-Pérez A, Pérez JA (2008) Selection of internal control genes for quantitative real-time RT-PCR studies during tomato development process. *BMC Plant Biol* 8: 131
